# Calorie-restriction treatment mitigates the aging in rat liver model

**DOI:** 10.1007/s10522-025-10245-8

**Published:** 2025-05-07

**Authors:** L. Urlandini, A. E. Leonetti, F. Conforti, A. Perri, D. Lofaro, G. Antonucci, M. Mandalà, S. Bossio, S. Di Agostino, V. Rago

**Affiliations:** 1 Department of Pharmacy, Health and Nutritional Sciences, University of Calabria, Rende, Italy; 2 Pathology Unit, Annunziata Hospital, 87100 Cosenza, Italy; 3 Department of Experimental and Clinical Medicine, Magna Græcia University of Catanzaro, Catanzaro, Italy; 4 Department of Biology, Ecology and Earth Science, University of Calabria, Rende, Italy; 5 Department of Health Sciences, Magna Græcia University of Catanzaro, Catanzaro, Italy; 6 University of Calabria, Department of Mathematics and Computer Science, Rende, 87036 Cosenza, Italy

**Keywords:** Aging, Caloric restriction, Liver, Inflammation, Fibrosis

## Abstract

The aging process promotes progressive impairment of homeostasis and the increase of the risk of disease and death. A major hallmark of the aging process is the systemic chronic inflammation which strongly contributes to the onset of aging-related diseases. In the liver, the aging condition drives the hepatocytes to develop a metabolic dysfunction-associated steatosis. Caloric restriction (CR) is a remarkable strategy to delay biological aging, occurring through several mechanisms. In this study we aimed to explore, employing an in vivo rat model, the impact of CR on aging-mediated liver inflammation markers. The experiments were performed on 14 male Sprague–Dawley rats (24 months old). At 18 months old, rats were allocated into two groups: the normal diet (ND) group was continued ad libitum diet, and the CR regimen group was fed a diet of the same chow restricted to 60% of the intake. All animals were sacrificed at 24 months old. Compared to the ND group, morphological examination of the liver revealed a lower level of fibrosis in the CR group, concomitantly with a reduced expression of key fibrotic markers, such as collagen I, fibronectin, and αSMA. Furthermore, CR improved the liver oxidative balance, as showed by the increased expression of two scavenging enzymes, SOD1/SOD. Moreover, we reported concomitant reduction of NLRP3 inflammasome signalling. Interestingly, CR significantly improved the signalling of key members of the nutrition-sensitizing affected by aging, AMPK/SIRT1/LKB1. Collectively our findings support the evidence on the metabolic benefits of CR about aging-related liver inflammation, by inducing a morphological improvement that mirrors the decrease in the expression of inflammatory molecular markers.

**Supplementary Information:**

The online version contains supplementary material available at 10.1007/s10522-025-10245-8.

## Introduction

In mammals, aging leads to the gradual loss of function and degeneration of multiple cells and tissues, thus representing the major risk factor for most chronic diseases, such as atherosclerosis and heart failure, osteoporosis, pulmonary insufficiency, renal failure, neurodegeneration and liver disease (Campisi 2013) (Trumble and Schneider-Crease 2020). Aging is characterized by an alteration of some metabolic processes, both in humans and rodents, which lead to an excess of visceral fat and the loss of muscle mass (Palmer and Jensen 2022). In terms of molecular mechanisms, aging is related to the dysregulation of cellular homeostasis detectable as an alteration of oxidative states and a low-grade chronic inflammation defined as inflammaging where the key factors involved are DNA damage, mitochondrial dysregulation, telomere dysregulation, NAD + loss, stem cell exhaustion and inhibition of autophagic mechanisms (Franceschi et al. 2000)*.* The set of biological and molecular processes that determine chronic inflammation in the aging process and in age-related diseases have been defined with the term senoinflammation (Noh et al 2024).

In the processes that lead to aging, although the liver seems to show greater physiological well-being than other organs if risk factors such as viruses, smoking and alcohol do not intervene, there is increasing evidence that hepatocytes show the classic signs of aging (Hunt et al. 2019).

The liver aging process is driven by genomic and epigenomic alterations and transcriptional reprogramming that contribute to the dysregulation of mitochondrial function and nutrient sensing pathways, leading to a low-grade inflammation stage and cellular senescence (Kim et al. 2015; Uyar et al. 2020). As a consequence of the aging process, the hepatocytes show proliferative arrest, concomitantly with altered production of specific molecules that support the pathological processes. The typical aging alterations highlighted in human patients and mice models derive from activated hepatic stellate cells (HSCs, also called Ito cells), which subsequently to the liver damage are the source of much of the extracellular matrix (ECM) production in the fibrogenesis process.

Recent data from a cross-sectional study have shown that biological aging accelerates liver fibrosis in patients with metabolically impaired fatty liver disease (MASLD) (Zhao et al. 2024). The dysregulation of hepatic energy metabolism contributes to common age-related diseases such as insulin resistance, diabetes mellitus, and metabolic dysfunction-associated steatosis liver disease (MASLD) (Georgieva et al. 2023). Over time, different pharmacological and non-pharmacological strategies have been proposed to counteract the effects of aging.

Among the non-pharmacological approaches, caloric restriction (CR) without malnutrition appears to be an effective therapeutic strategy, although most of the available studies are experimental on cohorts of patients affected by different types of diseases (López-Lluch and Navas 2016). Several studies converge on the idea that CR is an intervention that extends lifespan in mammals, delays the onset of age-related diseases, and delays several age-related degenerative processes. In recent years, many molecular mechanisms underlying CR over life span have been identified (Sun et al 2022; Duregon et al. 2023). At the molecular level, experimental data reported that CR is able to suppress the secretion of pro-inflammatory cytokines and chemokines, age-associated oxidative stress, to induce autophagic and epigenetic mechanisms, and to reverse systemic chronic inflammation (Yu and Chung 2001). Overall these findings suggest that CR could represent a promising tool for alleviating chronic inflammation and promoting antiaging effects (Noh et al. 2024).

The strongest evidence for the beneficial effects of calorie restriction on aging is reported in adipose tissue, kidney, heart and brain (Balasubramanian et al. 2017), while only recently the liver has started to become an interesting study model for mitochondrial activity and lipid metabolism, also because it is affected by other factors such as smoking and alcohol (Dorling et al. 2021; Chiang et al 2023; Ashiqueali et al. 2024). In this study we aim to evaluate the role of CR by monitoring the expression of key aging biomarkers in age-related liver damage using an animal model. We aim to investigate the morphological and molecular evidence indicating an age-dependent improvement of fibrosis in rat livers under a caloric-restricted feeding regimen.

## Materials and methods

### Animals

Experiments were performed on 14 aged male Sprague–Dawley rats (24 months old), which were housed in the animal care facility of the University of Calabria (Italy), in light (12:12 h light–dark cycle) and temperature (22 °C)-controlled rooms and had free access to food and water. The animals, at the age of 18 months old, the age at which signs of aging begin to appear (Phillips et al. 2010), were divided into two subgroups: Normal diet (ND) (n = 7) was continued ad libitum diet of a standard laboratory chow (Mucedola, diet 4RF21, Italy) and caloric restriction (CR) (n = 7) was fed a diet of the same chow restricted to 60% of the intake measured by weight in paired, control chow-fed rats, this level of restriction allows for better comparability of data between different studies as reported by (Ławniczak et al 2022; Prvulovic et al. 2022; Wrońska et al 2022). Food intake was recorded every other day while body mass was monthly. The age of sacrifice was 24 months old with inhalation of isoflurane 4% followed by cervical transection and immediately the organs were removed and placed in cold HEPES-physiological saline solution (HEPES-PSS). All procedures were conducted per the European Guidelines for the Care and Use of Laboratory Animals (Directive 2010/63/EU) and under Italian law. The project was approved by the Italian Ministry of Health (authorized n. 295/2016, of 18-02-2016), and by the Ethics Review Board of the University of Calabria. The haematological and biochemical parameters (Supplementary Table 1) of the animals have already been described in a previous study (Delwatta et al. 2018).

### Chemicals and antibodies

Unless otherwise indicated, reagents were purchased from Sigma Aldrich (Milan, Italy). The following primary antibodies were used: anti-Ki-67, anti-Lumican, anti-Fibronectin, anti-Collagen I, anti-Caspase 1, anti-NLRP3, anti-α-SMA, anti- NF-kB, anti p53, anti-SIRT1, anti-LKB1, anti-AMPK, pAMPK, anti-SOD1 and anti-SOD2, anti-PGC1α, anti-NRF2 (Santa Cruz Biotechnology, Santa Cruz, CA, USA), anti-phospho-CD44; (Cell Signalling Technology, Milan, Italy). Universal Biotinylated Horse IgG, was used as a secondary antibody and Diaminobenzidine chromogen (DAB) (Vector Laboratories, Burlingame, CA, USA).

### RNA extraction and RT-qPCR

Total RNA was extracted from tissues using Trizol (Thermo Fisher Scientific) according to the manufacturer’s protocol; the integrity and purity of the isolated total RNA was assessed using a Nanodrop Spectrophotometer. cDNA was synthesized from 1 µg of total RNA using the cDNA Reverse Transcription Kit (ThermoFisher, Waltham, MA, USA).

The qPCR was performed using Power Track SYBR Green Master Mix (Applied Biosystems), which contains the internal reference (ROX). Each qPCR reaction comprised: 10 μl 2 × SYBR Green PCR Master Mix, forward and reverse primer at optimized concentrations of 400 nM (final concentration). The following primers were used: NLRP3 forward GAGCTGGACCTCAGTGACAATGC, reverse ACCAATGCGAGATCCTGACAACAC; IL1β forward TGCTGTCTGACCCATGTGAG, IL1β reverse GTCGTTGCTTGTCTCTCCTTG; Caspase-1 forward AGGAGGGAATATGTGGG, reverse AACCTTGGGCTTGTCTT; β-actin forward CCCGCGAGTACAACCTTCT, β-actin reverse CGTCATCCATGGCGAACT. The qPCR reactions were done using the Quantstudio 3 (Thermofisher). The thermal profile was as follows: enzyme activation at 95 °C for 2 min followed by 40 cycles of denaturation at 95 °C for 5 s and combined annealing and extension at 60 °C for 30 s. Each qPCR experiment included triplicates of no-template controls and samples for all primers tested. We used β-actin to normalize the data. Relative expression was calculated using the comparative cross threshold (ΔΔCt) method as previously described (Livak and Schmittgen 2001).

### Liver tissues

Liver tissues were obtained after hepatectomy and each liver (prevalently the right lateral lobe) was divided into two parts, one was immediately fixed in 4% neutral buffered formalin and paraffin-embedded and carried out for histological and immunohistochemistry analysis, while the other was frozen and used for lipid staining and western blot analysis. Furthermore, we utilized two archived human liver samples, formalin-fixed and paraffin-embedded (FFPE), obtained from elderly patients (72 ND and 80 CR years old respectively) through the archives of the Pathological Anatomy Unit at Annunziata Hospital. These liver samples displayed features analogous to those observed in our experimental rat models. Specifically, one sample exhibited a metabolic dysfunction-associated steatotic liver disease (MASLD) (Kanwal et al. 2024), mirroring the characteristics of our ND group. The other sample, from a patient who practiced caloric restriction for religious reasons, showed normal morphofunctional liver characteristics comparable to those of our CR group.

### Histology and immunohistochemistry analysis

Histology and immunostaining of liver tissue sections were carried out at the Histology and Human Anatomy Laboratory, of the University of Calabria (Cosenza Italy). Briefly, de-paraffinized, hydrated serial sections of the liver were stained with hematoxylin and eosin, PAS, picrosirius red, and Masson’s trichrome, using standardized protocols. Additional serial sections were stained with antibodies previously indicated. Staining for all antibodies was optimized to ensure specificity with no background staining. Bound antibodies were detected using diaminobenzidinetetrahydrochloride (DAB) and sections were counter-stained with haematoxylin. Quantitative analysis of stained sections was performed as previously described (Perri et al. 2021). Absorption controls consisted of the primary antibodies pre-adsorbed with an excess of their purified blocking peptide, at 4 °C for 48 h.

### Imaging and scoring

Liver sections were visualized using an Olympus BX41 microscope and the images were taken withCSV1.14 software, using a CAM XC-30 for image acquisition. Immunoreactivity was scored as negative (0), weakly positive (1), moderately positive (2), positive (3), or strongly positive (4). For each sample, the most frequent score among the three independent observers was chosen. A minimum of 100 cells were evaluated in each slide. Seven serial sections were scored for each sample.

### Microarray data

We downloaded the gene expression profile of GSE102593 from the GEO database(Rusli et al. 2018). In this study, the authors reported the systemic and liver-specific responses caused by a diet switch to a medium-fat (MF) and caloric restriction (CR) diet in a 24-month-old life-long. Differential gene expression (DGE) analysis was performed using the GEO2R package (Bioconductor project). The gene set enrichment analysis was performed using the ShinyGO tool (Ge et al. 2020).

### Statistical analysis

Data were tested for normality using Shapiro–Wilk test at a significance level of p < 0.05. The intensity scores are presented as the median (IQR) of the sample groups (ND and CR) and compared using the Wilcoxon test. A p-value < 0.05 was considered statistically significant. All analyses were conducted with R (4.3.1). The Western blot and RT-qPCR data were analysed by the Mann–Whitney test and unpaired Student’s test using GraphPad/Prism version 5.01 statistical software (SAS Institute, Abacus Concept Inc., Berkeley, CA, USA). Data are expressed as means ± standard error (SE). A p-value of < 0.05 was considered statistically significant.

## Results

### Caloric restriction mitigates liver fibrosis aging-related

The ND rats maintained an ad libitum diet showing a median body weight of 9 gr (IQR − 65.1, 16.7) respect to CR rats that showed a median decrease of − 229 gr (− 282, − 167; p = 0.001).

**Fig. 1:**
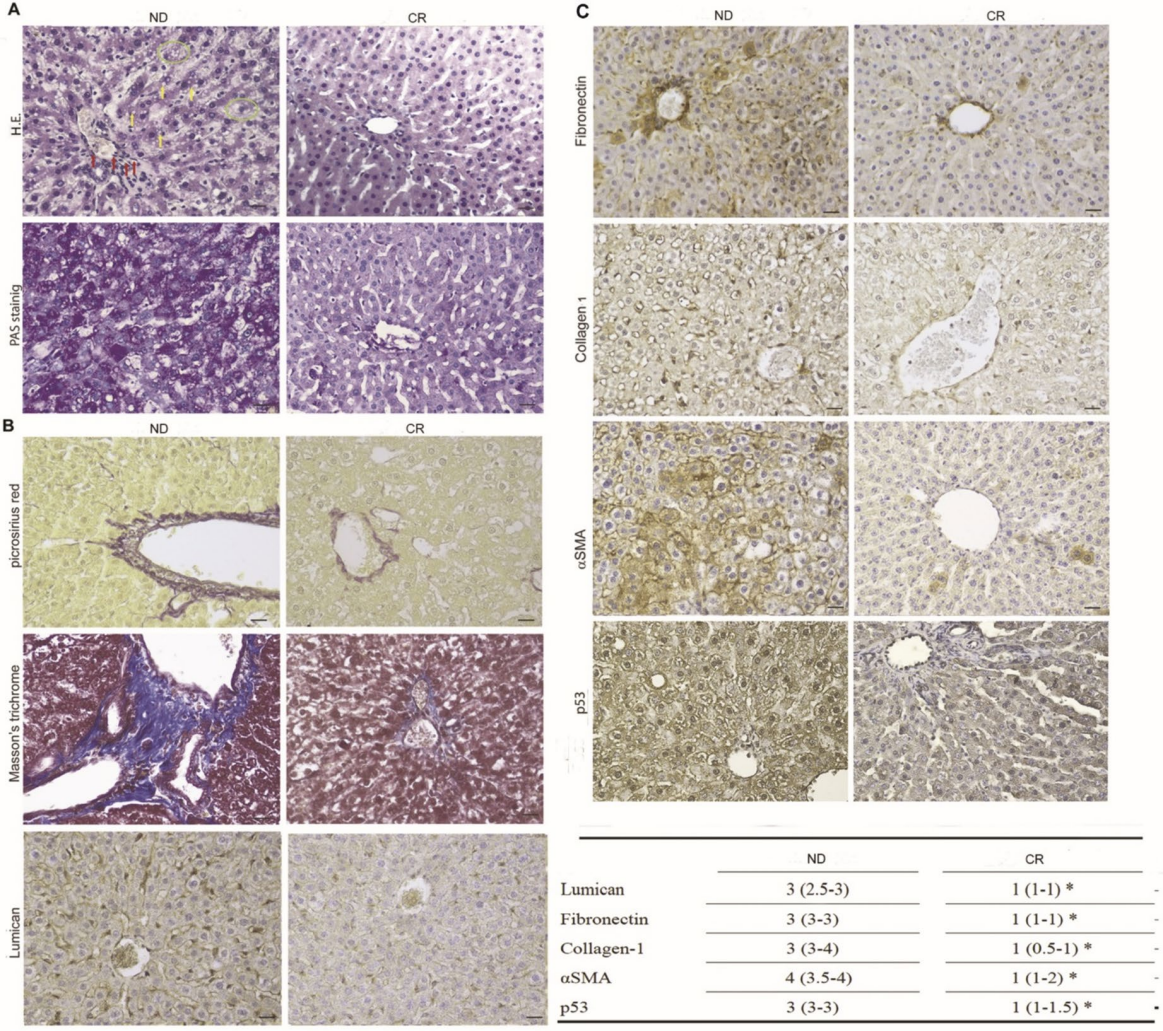
**A** Haematoxylin & Eosin (HE) and Periodic acid–Schiff (PAS) staining, **B** Picrosirius red, Masson’s trichrome and Lumican expression, **C** Immunohistochemical expression of p53 and fibrosis markers (Fibronectin, Collagen I, and αSMA) in ND (left panel) and CR (right panel) rats. Scale bars: 25 µm. Yellow arrows: active hepatocytes and Kuppfer cells enriched with vacuoles; red arrows: inactive Kuppfer cells located around the sinusoids; green circles: kupffer cells with vacuolated cytoplasm, grouped in clusters rich in macrophages or other inflammatory cells such as lymphocytes or plasma cells. The lower table shows the immunoreactivity score median (IQR); *p < 0.05 vs ND

The Haematoxylin & Eosin (HE) staining highlighted in the liver of ND rats a marked micro–macro-vesicular steatosis MASLD with feathery degeneration of the hepatocytes (ballooning degeneration), and focal chronic inflammation accompanied by an evident expansion of the portal spaces due to chronic inflammation with short fibrotic septum (Israelsen et al. 2024). Conversely, in the liver of CR rats, HE staining showed hepatic parenchyma arranged in trabeculae with well-represented sinusoidal spaces, hepatocytes of normal shape and size free of steatosis, and portal spaces regularly arranged between the lobules (Fig. 1A).

Hepatic fibrosis, the common endpoint of several chronic liver injuries (Kmieć 2001) is characterized by the increase of collagen in the matrix, resulting in ‘scar’ tissue (Reeves and Friedman 2002). In our study, fibrosis was assessed by specific staining which directly marks the extracellular matrix. Aging-related fibrosis in the ultrastructure of ND hepatocytes is evident from the accumulation of PAS-positive inclusions representing misfolded fibers and from the high number of vacuoles within the cells compared to CR liver tissues (Fig. 1A) (Łysek-Gładysińska et al. 2021). The Sirius Red staining highlighted that the liver tissues of the ND group were positive with a 3- to fourfold increase compared to the CR group. The same results were obtained after Masson’s trichrome staining (Fig. 1B). We further evaluated Lumican expression, an extracellular matrix proteoglycan that contributes to liver fibrosis by maintaining collagen fibril stability (Krishnan et al. 2012). In ND liver samples, Lumican expression resulted at higher levels compared to the CR group (Fig. 1B). Furthermore, the liver tissues of CR rats showed a significantly lower expression of different markers of fibrosis, such as Fibronectin, Collagen 1, and αSMA, compared to that detected in the samples of ND rats (Fig. 1C). Finally, we evaluated the expression of the tumor suppressor p53, that although in the liver upon acute injury it exerts anti-inflammatory and antifibrotic effects, in the long time its chronic activation promotes liver fibrosis (Yu et al. 2022). In line with the above-reported findings, in CR samples we observed a significant reduction of nuclear p53 expression compared to liver tissues of the ND group (Fig. 1C). Overall, these results indicated that CR significantly reduces morphological features and histochemical marker of age-related fibrosis.

### Caloric restriction decreases inflammatory markers and increases antioxidant markers in aged liver tissue

**Fig. 2:**
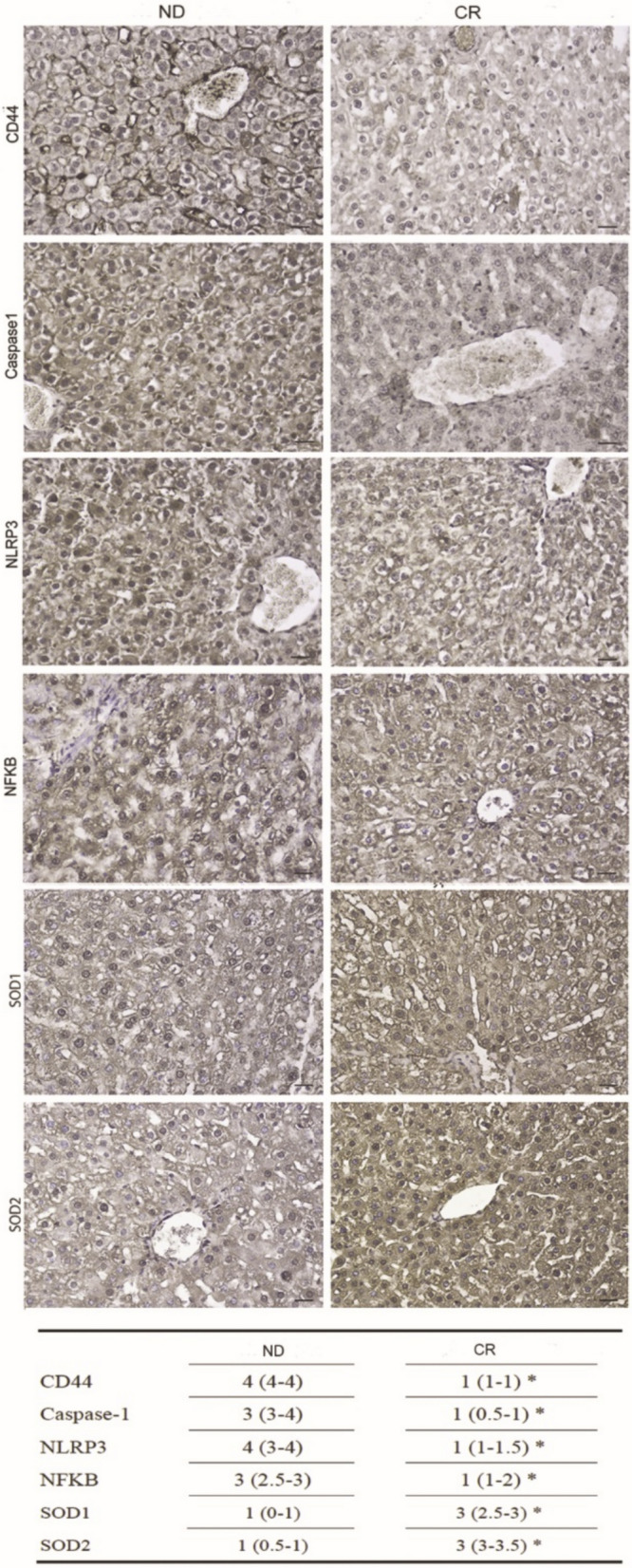
Immunohistochemical expression of pro-inflammatory markers (CD44, Caspase-1, NLRP3, NFkB) and anti-oxidant enzymes (SOD1, SOD2) in ND (left panel) and CR (right panel) rats Lower table shows immunoreactivity score median (IQR); *p < 0.05 vs ND. Scale bars: 25 µm

Oxi-inflammaging is considered a hallmark of aging, that strongly contributes to the onset and progression of aging-related diseases (Li et al. 2023). CD44 is expressed by active T lymphocytes and it is a cell–cell adhesion molecule involved in inflammatory cell infiltration (McDonald and Kubes 2015). Accordingly, the IHC analysis revealed that the liver tissue from CR rats exhibited a lower expression of CD44 compared to the CR group (Fig. 2).

Furthermore, we have investigated the expression of the NLRP3 inflammasome pathway which is involved in the activation of DNA damage response and the mitochondrial dysfunction of the senescence-related inflammatory process (Franceschi et al. 2000). Interestingly, by IHC we observed a significant reduction of NLRP3 expression in CR group concomitantly with its downstream effector Caspase 1 and of the transcription factor NFkB, which is involved in the transcription of both NLRP3 and its downstream proinflammatory interleukins (Fig. 2).

During aging, exposure to reactive oxygen species increases the incidence of liver damage, especially in female (Aydin et al. 2010). Therefore, even if the programming of aging mechanisms it may be gender specific, our results demonstrated by expression of two key cytoplasmic antioxidant enzymes, SOD1 and SOD2, significantly higher expression of both SOD1 and SOD2 in CR tissues compared to ND liver tissues suggesting that CR counteracts oxidative stress in old liver (Fig. 2).

We further evaluated by IHC the expression of two key cytoplasmatic antioxidant enzymes, SOD1 and SOD2. We observed a significantly higher expression of both SOD1 and SOD2 compared to ND liver tissues, strongly suggesting that CR counteracts oxidative stress in old liver (Fig. 2).

### Caloric restriction restores the activation of AMPK signalling

**Fig. 3:**
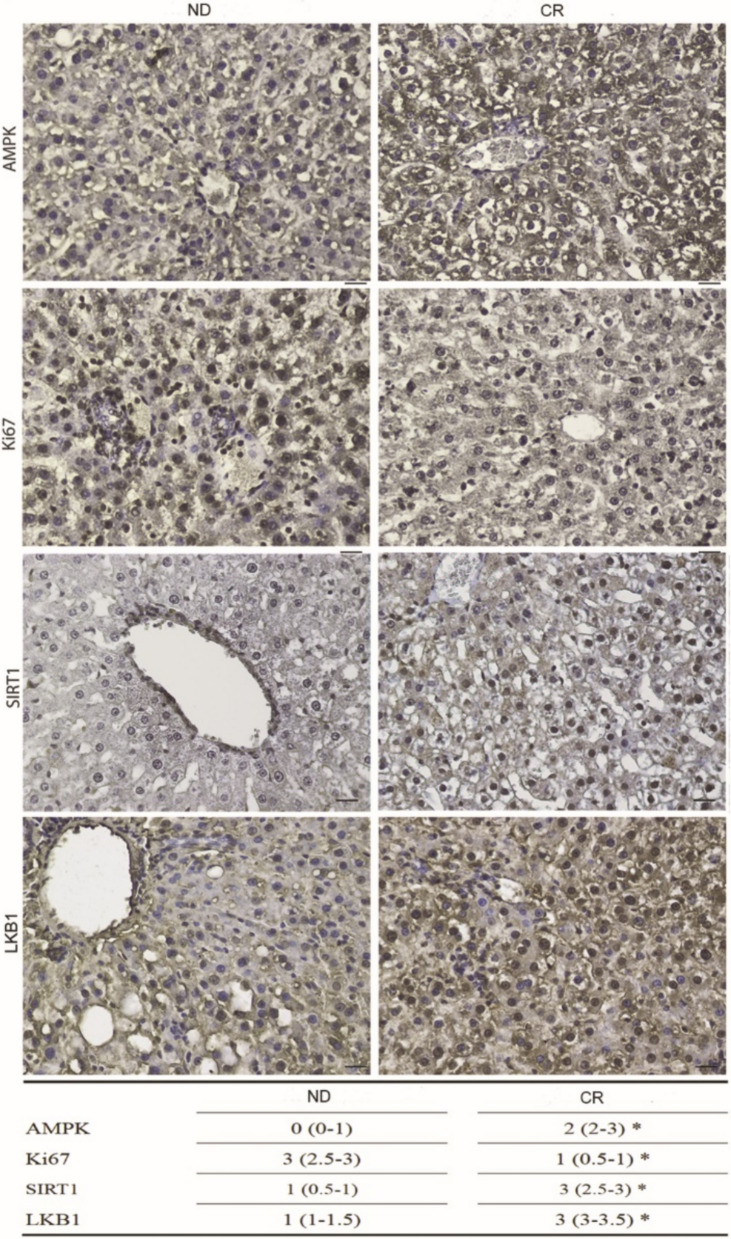
Immunohistochemical expression of AMPK signaling and Ki67 in ND (left panel) and CR (right panel) rats Scale bars: 25 µm. Lower table shows the immunoreactivity score median (IQR); *p < 0.05 vs ND

Lipid metabolism disorders are the main causes of the onset and progression of various liver diseases caused by a high-fat and ethanol diet. The AMP-activated kinase (AMPK) signalling pathway plays an important role in improving lipid metabolism disorders and for this reason it has become a target for the development of targeted therapies to treat type 2 diabetes and obesity (Fang et al. 2022). Many studies reported that AMPK activation decreases during aging and that the lifespan effect promoted by CR is linked to the activation of Silent information regulator 1 (SIRT1), AMPK, LKB1 which directly phosphorylates and activates AMPK and peroxisome proliferator-activated receptor gamma coactivator 1-alpha (PGC-1α) (Woods et al. 2017). Accordingly, we investigated their expression by IHC in our old liver model. We found that liver tissues of CR rats exhibited higher AMPK, SIRT1, and LKB1 staining, concomitantly with a lower expression of the proliferative marker Ki-67 compared to ND liver tissue (Fig. 3).

These expression analyses on old liver tissues from ND and CR rats suggest that CR contributes to restoring AMPK signaling and decreasing the fibrotic morphological features of the liver.

### Effects of caloric restriction on the molecular and biochemical expression of inflammation-related markers in old liver

**Fig. 4:**
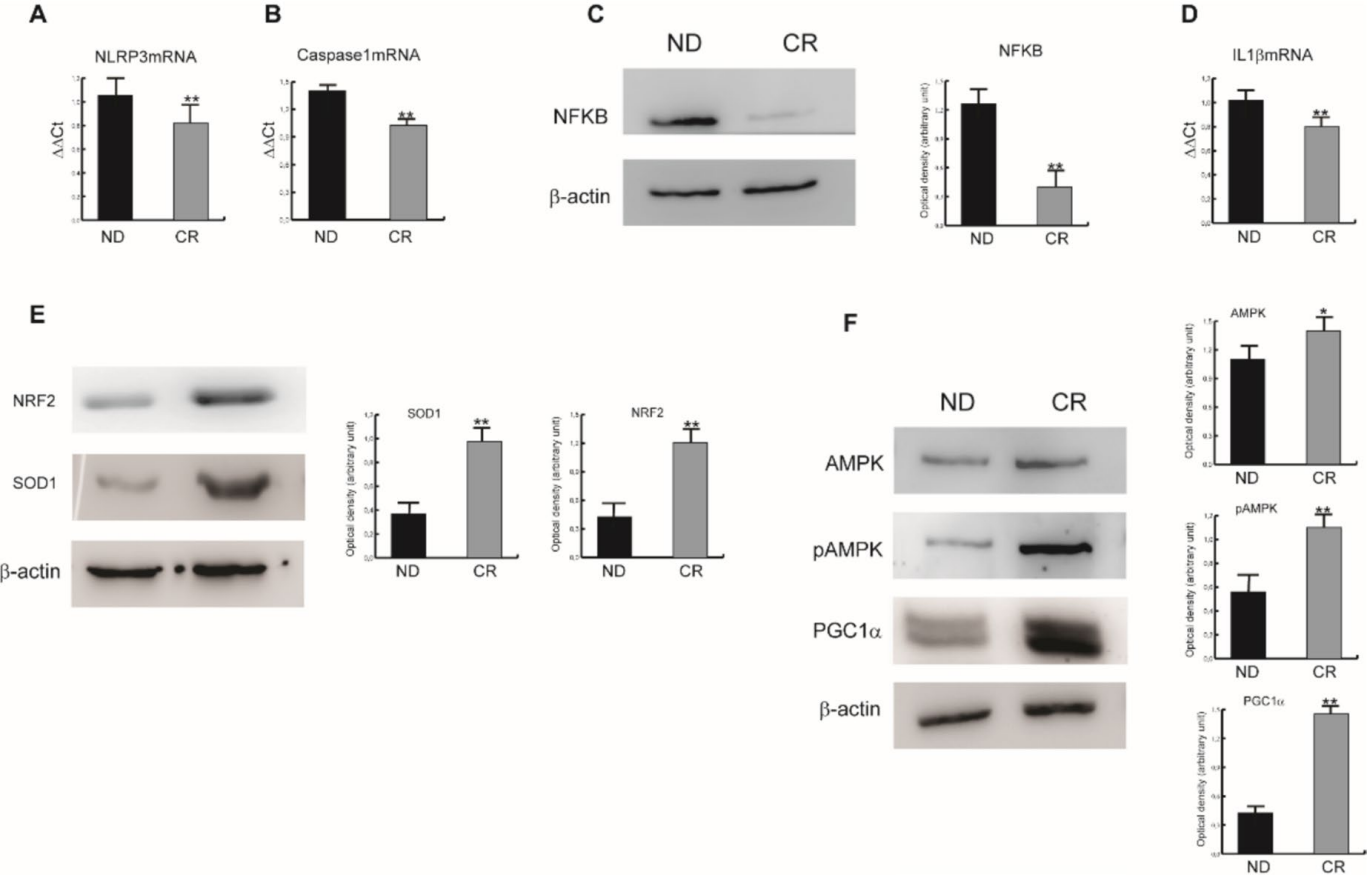
Representative Real‐time PCR analysis for NLRP3 (**A**), Caspase‐1 (**B**), and IL‐1β (**D**) in the liver tissues from ND and CR rats; ** = p < 0.05. Bars represent the mean ± SE of 3 experiments. Representative Western blotting of NFkB (**C**), SOD-1, and NRF2 (**E**) and total AMPK, pAMPK, and PGC1α (**F**) protein expressions in liver tissue samples of ND and CR rats. Protein loading was verified by using the anti-β-actin antibody. Data are means ± SE of three determinations for each animal (n = 3). Statistical differences were evaluated by the Mann–Whitney U-test (** p < 0.05). The columns represent the band intensities, evaluated in terms of arbitrary densitometric units, and presented as the mean ± SEM

After the evidence obtained by IHC analysis, we moved to investigate the expression of the key molecules of the pathways of our interest at a molecular and biochemical level. In order, we highlighted a significant decrease in NLRP3 protein expression in the CR group in parallel with its downstream effector Caspase 1, and the reduction of protein and mRNA levels of the transcription factor NFkB, which is involved in the transcription of both NLRP3 and its downstream proinflammatory interleukins (Fig. 4A–C). High IL-1β/IL-6 and NLRP3 levels drive liver fibrosis in experimental models of non-alcoholic fatty liver disease (NAFLD) mice (Barbier et al. 2019). Along with the data shown, the livers of CR regimen rats showed a decrease in IL-1β transcript expression (Fig. 4D).

In line with the results described above we also investigated the expression of NRF2, the master regulator of cellular redox homeostasis whose levels decrease with the aging process. We found that protein NRF2 level increases in the CR group to activate genes related to detoxification reactions (Fig. 4E). Furthermore, NRF2 activation is responsible for the suppression of inflammation by NF-kB, which decreases, as observed in Fig. 4C.

By IHC, we documented that CR was able to rescue the energy sensing network by upregulation of AMPK, SIRT1 and LKB1 (Fig. 3). Here we assessed that CR induced not only the level of AMPK protein but also its phosphorylation levels indicating its activation in the pathway (Fig. 4F). SIRT1 is an enzyme that mediates NAD + -dependent deacetylation of target substrates and can directly induce the transcriptional activity of PGC-1α which transcribes genes implicated in mitochondrial biogenesis and respiration rates (Cantó and Auwerx 2009). Accordingly, we reported the increased PGC-1α protein expression in the liver tissues of CR group (Fig. 4E).

Overall, these data provide strong evidence that the CR regimen is able to influence an intricate framework of metabolic sensors that collectively tend to mitigate liver inflammation caused by the aging process.

**Fig. 5:**
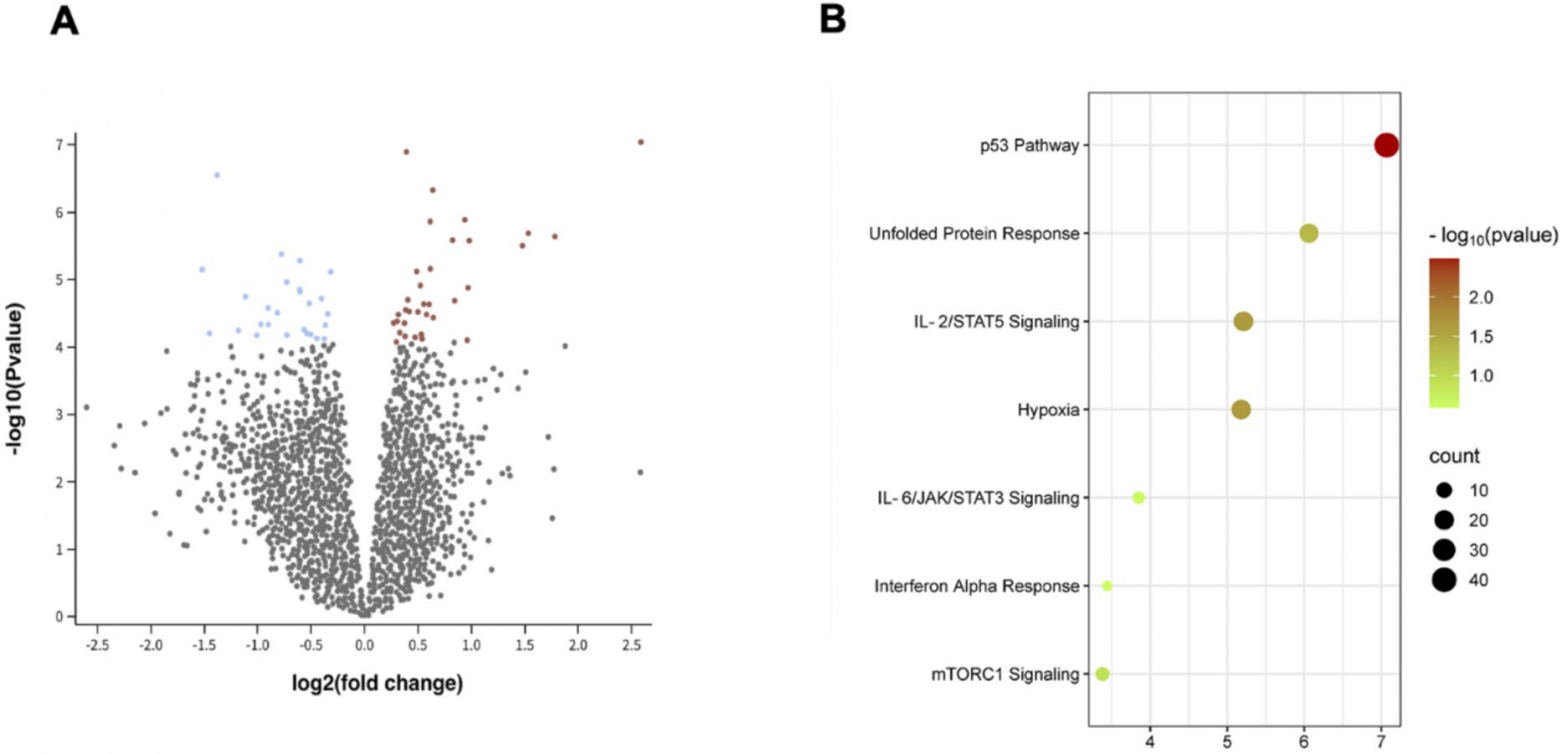
Differential analysis derived from the expression profiling by array GSE102593. **A** Volcano plot displaying 34 significantly upregulated (Red) and 26 downregulated (Blue) genes. x-axis: -log2(p-value) which approximates fold change (FC) obtained from the Wald test. Y-axis: -log10 (p-value) of significant genes. The genes list is available in Table 1. **B** A bubble plot illustrating a selection of REACTOME-enriched pathways obtained by ShinyGO with a False Discovery Rate (FDR) (bubble sizes represent Fold Enrichment). The enriched analysis is carried out from DEGs in (**A**), between old liver tissues of mice normally fed and mice subjected to caloric restriction

To further give robustness to the data we have described so far, we considered a public microarray dataset (GSE102593) where livers taken from 28-month-old mice fed with water and food ad libitum, (control), calorie restriction diet (CR) and medium fat diet were studied (Rusli et al. 2018). Here, we analyzed the CR group versus the normally fed group. Notably, from the altered pathways derived from the analysis of differentially expressed genes (DEGs) among the old liver tissues of CR mice compared to the control group (Fig. 5A**; **Table 1), we reported as significantly modulated the signalling of p53, interleukin-2 and its downstream target STAT5 (Fig. 5B). These pathways exert their effect on many aspects of immune function and are supported by the activity of AMPK in aged mice (Pokhrel et al. 2022), hypoxia pathway as the sensor of the redox imbalance and oxygen alteration, and UPR signalling as expected, correlated with the fibrosis state of aging (Fig. 4B). Interestingly, CR can affect the JAK/STAT3 pathway, which includes more than 50 molecules including hormones, interferons (IFNs) and interleukins (ILs), which governs events including haematopoiesis, immune response, tissue repair, inflammation, apoptosis and adipogenesis (Fig. 5B**)**.

**Table 1:** analysis of differentially expressed genes (DEGs) among the old liver tissues of CR mice compared to the control group

ID	Gene symbol	Gene title	log2 (fold change)	−log10 (P value)
10,404,595	Ppp1r3g	Protein phosphatase 1, regulatory (inhibitor) subunit 3G	2.588	7.039
10,364,093	Derl3	Der1-like domain family, member 3	1.782	5.638
10,561,055	Ceacam2	Carcinoembryonic antigen-related cell adhesion molecule 2	1.534	5.688
10,585,037	Bud13	BUD13 homolog	1.479	5.503
10,542,880	Mettl20	Methyltransferase like 20	0.98	5.578
10,529,979	Ppargc1a	Peroxisome proliferative activated receptor, gamma, coactivator 1 alpha	0.969	4.879
10,494,039	Lingo4	Leucine rich repeat and Ig domain containing 4	0.961	4.101
10,585,022	Zpr1	ZPR1 zinc finger	0.939	5.886
10,481,634	Slc25a25	Solute carrier family 25 (mitochondrial carrier, phosphate carrier), member 25	0.844	4.687
10,523,111	–	–	0.824	5.586
10,492,231	Med12l	Mediator complex subunit 12-like	0.643	4.437
10,585,428	Dnaja4	DnaJ heat shock protein family (Hsp40) member A4	0.639	6.327
10,427,997	Ankrd33b	Ankyrin repeat domain 33B	0.616	5.16
10,365,344	Tcp11l2	t-complex 11 (mouse) like 2	0.615	5.862
10,428,004	Ankrd33b	Ankyrin repeat domain 33B	0.604	4.633
10,529,068	Slc30a3	Solute carrier family 30 (zinc transporter), member 3	0.58	4.483
10,405,779	Mir23b	microrna 23b	0.554	4.638
10,343,829	–	–	0.538	4.122
10,378,637	Scarf1	Scavenger receptor class F, member 1	0.531	4.19
10,470,125	Ccdc183///Fcna	Coiled-coil domain containing 183///ficolin A	0.523	4.913
10,585,390	Sln	Sarcolipin	0.5	4.522
10,586,700	Rora	RAR-related orphan receptor alpha	0.488	5.12
10,450,533	Vars2	Valyl-tRNA synthetase 2, mitochondrial	0.473	4.144
10,515,242	Nsun4	NOL1/NOP2/Sun domain family, member 4	0.418	4.528
10,481,759	Slc2a8	Solute carrier family 2, (facilitated glucose transporter), member 8	0.405	4.701
10,517,948	Spen	SPEN homolog, transcriptional regulator (Drosophila)	0.392	6.895
10,451,167	Tmem63b	Transmembrane protein 63b	0.385	4.55
10,572,815	Nwd1///Tmem38a///Nwd1///Tmem38a	NACHT and WD repeat domain containing 1///transmembrane protein 38A///NACHT and WD repeat domain containing 1///transmembrane protein 38A	0.379	4.159
10,475,144	Ganc///Capn3	Glucosidase, alpha; neutral C///calpain 3	0.374	4.355
10,498,319	Serp1	Stress-associated endoplasmic reticulum protein 1	0.33	4.214
10,591,281	Col5a3	Collagen, type V, alpha 3	0.317	4.482
10,434,003	Crkl	v-crk avian sarcoma virus CT10 oncogene homolog-like	0.306	4.382
10,504,106	Gm13305///Gm2002///Il11ra2///Il11ra1	Predicted gene 13305///predicted gene 2002///interleukin 11 receptor, alpha chain 2///interleukin 11 receptor, alpha chain 1	0.298	4.076
10,563,260	Snrnp70	Small nuclear ribonucleoprotein 70 (U1)	0.272	4.357
10,381,272	Cntnap1	Contactin associated protein-like 1	− 1.52	5.15
10,341,310	–	–	− 1.452	4.203
10,445,325	Rcan2	Regulator of calcineurin 2	− 1.38	6.55
10,522,788	Stap1	Signal transducing adaptor family member 1	− 1.18	4.246
10,464,772	2010003K11Rik	RIKEN cDNA 2010003K11 gene	− 1.115	4.747
10,478,525	Wfdc2	WAP four-disulfide core domain 2	− 1.009	4.175
10,344,489	–	–	− 0.97	4.337
10,540,059	Slc41a3	Solute carrier family 41, member 3	− 0.903	4.58
10,360,382	Mnda///Ifi204	Myeloid cell nuclear differentiation antigen///interferon activated gene 204	− 0.901	4.334
10,581,813	Mlkl	Mixed lineage kinase domain-like	− 0.816	4.508
10,411,373	Hexb	Hexosaminidase B	− 0.778	5.379
10,490,872	Lrrcc1	Leucine rich repeat and coiled-coil domain containing 1	− 0.729	4.963
10,542,911	Samd9l	Sterile alpha motif domain containing 9-like	− 0.727	4.177
10,449,303	Bak1	BCL2-antagonist/killer 1	− 0.607	4.851
10,385,271	Ccng1	Cyclin G1	− 0.606	5.282
10,508,917	Aim1l	Absent in melanoma 1-like	− 0.604	4.823
10,421,258	Pdlim2	PDZ and LIM domain 2	− 0.568	4.26
10,539,472	Nagk	N-acetylglucosamine kinase	− 0.537	4.202
10,466,947	Ermp1	Endoplasmic reticulum metallopeptidase 1	− 0.518	4.647
10,380,739	Osbpl7	Oxysterol binding protein-like 7	− 0.503	4.184
10,529,584	Man2b2	Mannosidase 2, alpha B2	− 0.446	4.129
10,473,847	Ddb2///Acp2	Damage specific DNA binding protein 2///acid phosphatase 2, lysosomal	− 0.404	4.717
10,387,525	Mpdu1	Mannose-P-dolichol utilization defect 1	− 0.376	4.122
10,468,311	Sh3pxd2a	SH3 and PX domains 2A	− 0.367	4.326
10,583,697	Smarca4	SWI/SNF related, matrix associated, actin dependent regulator of chromatin, subfamily a, member 4	− 0.344	4.491
10,559,261	Cd81	CD81 antigen	− 0.317	5.116

### Caloric restriction mitigates MASLD features in aged human liver samples

To explore our findings in human liver tissue samples, we considered two cases of elderly patients (72 and 80 years old) where one sample exhibited a metabolic dysfunction-associated steatotic liver disease (MASLD) mirroring the characteristics of our ND group in the rat model, and the other sample was derived from a patient who practiced caloric restriction for religious reasons, similarly to the CR group.

**Fig. 6:**
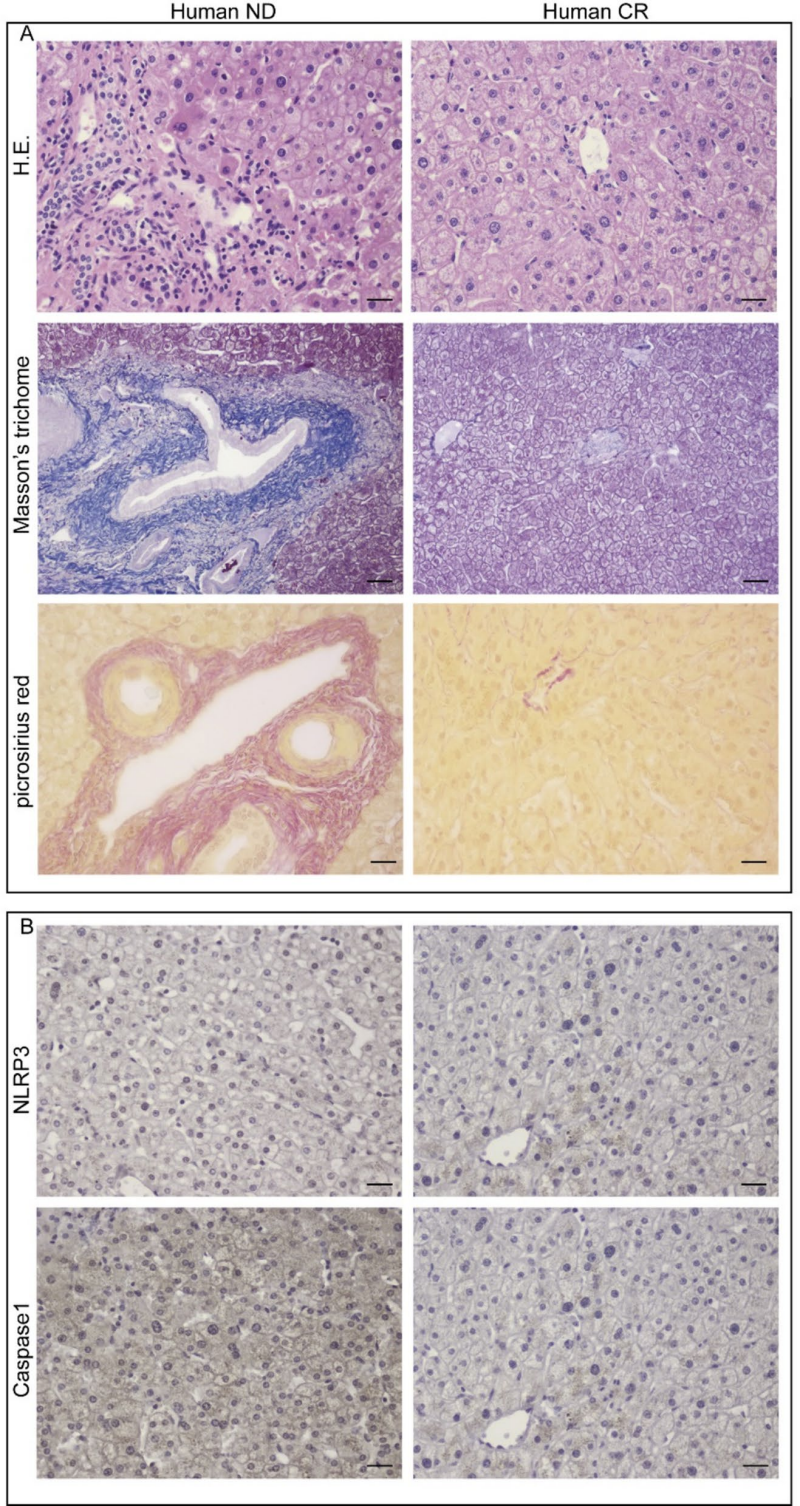
Evaluation of fibrosis and inflammatory state in liver human samples. **A** HE, Picrosirius red, and Masson’s trichrome stainings in ND (left panel) and CR (right panel) subjects. **B** Immunohistochemical expression of pro-inflammatory markers NLRP3 and Caspase-1in ND and CR human liver tissue. Scale bars: (HE, NLRP3, and Caspase-1) 25 µm; Picrosirius red and Masson’s trichrome 50 µm

The morphological and IHC analyses conducted on these human liver tissue samples revealed micro–macro-vesicular MASLD and fibrotic infiltrates in the sample, which exhibited tissue characteristics similar to our ND experimental group. In contrast, the other sample presented morphofunctional characteristics similar to that detected in the CR experimental group. In these samples, fibrosis was evaluated using H&E, Masson’s Trichrome, and picrosirius red stains, while the inflammatory state was highlighted through the immunoexpression of NLRP3 and its downstream effector, Caspase 1. The results obtained revealed a clear reduction of the fibrotic infiltrate in the patient’s liver, with no signs of MASLD (Israelsen et al. 2024). Additionally, in the same sample, we observed a reduction in the expression of NLRP3 and Caspase-1, compared to tissue with clear signs of MASLD (Fig. 6).

Even if we have considered individual cases, these results strongly suggest that these human conditions are similar to what we observed in our animal models where CR counteracts liver fibrosis and inflammation associated with aging. However, these findings need to be confirmed in a larger sample of human liver tissues, to ensure the validity.

## Discussion

The data highlighted by this in vivo study strongly supported the hypothesis that CR efficaciously counteracts hepatic pathological changes related to aging, such as fibrosis, and improves oxi-inflammaging and nutrient-sensing signalling.

Aging is a multifactorial process characterized by systemic low-chronic inflammation, which is accompanied by cellular senescence and organ dysfunction, leading to the onset of many aging-related diseases (Li et al. 2023). A large body of evidence provided that CR can modulate the hallmarks of aging, such as genomic instability, loss of proteostasis, deregulating nutrient sensing, telomere length, and altered intercellular communication, resulting in lifespan extension (Grande de França et al. 2023).

Most of the studies on CR and aging concern the observation of for example the slowing down of brain aging, the metabolic change in adipose tissue and in the cardiovascular system (Fontana 2008; Suchacki et al. 2023; Wilson et al. 2024). Studies on the effects of CR on old or diseased livers are still few, and many molecular and biochemical pathways are still unexplored.

The liver is an organ with complex and finely regulated metabolic processes which play a crucial role in maintaining the homeostasis of the entire organism (Rui 2014; Kim et al. 2015). Liver fibrosis is a pathological condition characterized by the excessive accumulation of scar tissue in the liver. Liver fibrosis is promoted by several events, such as excessive ECM and collagens accumulation, immune cells infiltration, and hepatocytes senescent-induced activation of hepatic stellate cells that secretes many pro-fibrotic and pro-inflammatory factors (Li et al. 2022, 2023).

Some recent studies have focused on the effects of short- and long-term caloric restriction, both in animal models and humans, assessing biochemical parameters in blood, muscle mass, and adipose tissue (Sato et al. 2017; Dorling et al. 2021; Schädel et al 2023). In non-obese individuals, 2 years of CR showed overall body improvement and improvement in several liver biomarkers, with potentially greater improvements in men (Dorling et al. 2021). Interestingly, Sassone-Corsi group demonstrated that CR can reprogram circadian metabolism at the transcriptional level by improving liver metabolism in aged mice (Sato et al. 2017).

The staining techniques used in our study to detect collagen fibers and ECM deposition revealed that CR strongly counteracts liver fibrosis. CR rat samples showed a significant reduction in “scar” liver tissue compared to the ND liver, concomitantly with a lower expression of key fibrotic markers, such as fibronectin and α-SMA. These results suggested that CR can mitigate age-related liver fibrosis by reducing the secretion of profibrotic factors.

It has been reported that NLRP3-inflammasome signalling activation is closely associated with age‑related liver injury and liver fibrosis and that excessive ROS accumulation activates the NLRP3 inflammasome in the liver during the process of aging, eventually leading to aging‑associated liver disease (Gallego et al. 2020; Li et al. 2021). Interestingly, some of the effects displayed by the CR are believed to be dependent by innate immune cell modulation, as CR resulted associated with a significant reduction of the metabolic and inflammatory activity of the circulating monocyte pool and blockage of NLRP3 inflammasome activation, leading to an improvement of the systemic inflammatory profile (Youm et al. 2015; Procaccini et al. 2023). The results emerging from our analysis suggest that CR may reduce the recruitment of inflammatory and immune cells in the liver tissue since in CR rats we observed a decreased expression of the adhesion molecule CD44 and NLRP3 signalling pathway compared to the ND group.

The level of ROS increases with age according to the mitochondrial dysfunction, whereas the activity of ROS scavenging enzymes decreases (Son and Lee 2019). Interestingly, in the CR liver tissues, we detected high expression of two key scavenging enzymes, SOD1 and SOD2, suggesting that CR also improves the liver oxidative balance. These results agree with the study of La Russa and colleagues demonstrating that CR decreases the plasmatic oxidative stress in the same animal model (La Russa et al. 2020).

Some authors reported that aged tissues have a deficiency in the sensitivity of AMPK activation mainly caused by chronic low-grade inflammation. AMPK stimulates the SIRT1 signalling pathway that, in turn, regulates cellular energy metabolism, many components of cell survival, cell proliferation, inflammation, and stress resistance (Salminen et al. 2016).

Furthermore, SIRT1 increases the activity of LKB1, an upstream activator of AMPK. Therefore, the positive feedback loop between SIRT1 and AMPK exerts an important role in maintaining the function also of the other AMPK-activated signaling pathways (Salminen and Kaarniranta 2012). CR has been seen to induce SIRT1 activity, leading to an increase in cellular stress resistance, which is a well-assessed defence mechanism against the aging process. Therefore, the positive feedforward loop may amplify the response to CR (Le Bourg 2009). In line with the above-reported evidence, a further interesting finding emerging from our study is that CR significantly increases the liver expression of AMPK, SIRT1, and LKB1, suggesting that the nutritional intervention can improve the signalling of key molecules of the nutrition-sensitizing affected by aging. To further support our results, we interrogated a microarray dataset (GSE102593) where different groups of aged mice were fed ad libitum, with a medium-fat diet and a calorie-restricted diet (Rusli et al. 2018). Our analysis of DEGs in aged mice livers subjected to CR showed significant modulations of pathways related to immune defence, cellular redox balance, and fibrosis status of cell signalling aging compared to control.

Metabolic-associated fatty liver disease (MASLD) is the most common cause of chronic liver disease, affecting approximately 25% of the world’s population, resulting in fibrosis, and it is the leading cause of liver-related mortality (Chan et al. 2023). Although many risk factors are associated with MASLD, the mechanisms underlying its onset and progression are not well clarified yet. The increased risk of developing MASLD during aging, strongly suggests that the interplay between the genetic predisposition and the aging factors have a critical role in MASLD development (DiLeo et al. 2023). Furthermore, some studies suggest that dietary education, caloric restriction, and exercise are the cornerstones for the management of MASLD (Georgieva et al. 2023)*.* Given this evidence and to further strengthen our results, we investigated the histological features and the expression of fibrosis and inflammation markers in the liver tissue of an old subject undergoing CR, compared to those observed in an old subject not following any dietary restrictions. Interestingly, our investigation showed results in line to those obtained by our in vivo study. Specifically, the liver tissue of the subject submitted to CR did not exhibit morphological markers of MASLD, fibrosis, and inflammation that, conversely, were detectable in the sample of the subject following no diet restriction. Undoubtedly, this last result needs to be further confirmed by increasing the sample size, but solid data from clinical trials already demonstrate that lifestyle modifications, including caloric restriction, attenuate age-related liver features (Sun et al 2025).

## Conclusions

Overall, the results emerging from our study reinforced the important beneficial effects of CR on the liver, highlighting its impact on several age-related signalling. CR is considered one of the most promising dietary interventions to extend lifespan in humans. ROS, although considered dangerous molecules in redox metabolism, can contribute to other cellular processes (Atayik and Çakatay 2023). In fact, their optimal level modulates signal transduction such as that mediated by NRF2, acting as second messengers for redox-sensitive cascades. Consequently, redox-dependent alterations may influence the age-associated decline in liver function. It is known that aging is also associated with a reduction in Nrf2 activity (Cuadrado 2016), in fact Nrf2 activation induces its translocation to the nucleus where it activates the expression of target genes that contain the ARE DNA regulatory sequence in their promoter region (Jaiswal 2004). This Nrf2/ARE pathway is modulated by KEAP1, which in basal conditions, acts as an Nrf2 repressor, blocking its nuclear translocation. Although alteration of redox homeostasis occurs in both aging and impaired liver regeneration, the associative mechanisms are not clearly defined. Of note, antioxidants are not effective in slowing down liver senescence. Further investigations are needed to define the reciprocal redox-dependent molecular pathways involved in both aging and the decline of liver regeneration. Our results show that CR induces anti-aging effects, as also suggested by the activity of calorie restriction mimetics (CRMs), a large group of natural compounds that provide similar molecular and biochemical effects to CR by inducing autophagy (Atayik and Çakatay 2023). CRMs regulate redox signaling by enhancing antioxidant defense mechanisms via activation of the Keap1/Nrf2/ARE system and by inhibiting ROS formation via attenuation of mitochondrial dysfunction.

However, from a practical perspective for the application of CR in humans, further studies are needed to better clarify the molecular mechanisms of CR and understand the potential interference of clinical confounders.

## Limitations

Several limitations in our study must be recognized. First, the duration of the CR regimen study was six weeks, and the long-term effects of CR need to be further studied. Second, in the continuation of our study we would like to perform a transcriptomic analysis on aged liver tissues from ND and CR mice to find novel pathways that can be modulated by CR and identify molecular targets that could be modulated by molecules already in use in therapies whose purpose is to alleviate the symptoms of fibrotic suffering of the aged liver. Finally, we believe that the analyses performed on the two human tissues, although not significant from a numerical point of view, it was not the main purpose of the study to create a cohort of patients, were useful in corroborating our study rationale.

## Supplementary Information

Below is the link to the electronic supplementary material.

Supplementary file1 (DOCX 18 KB)

## Data Availability

No datasets were generated or analysed during the current study.

## Code availability

There is no custom computer code or algorithm used to generate the results reported in the Manuscript.
