# Supplementary material for: Calorie-restriction treatment mitigates the aging in rat liver model
Source: Biogerontology. 2025 May 7;26(3):108. doi: 10.1007/s10522-025-10245-8 (PMC12058891; doi:10.1007/s10522-025-10245-8)
Supplement: Supplementary file 1 — Supplementary file1 (DOCX 18 KB) [file 10522_2025_10245_MOESM1_ESM.docx]

**Supplementary Table 1**

| **Parameter** | **Normal diet** | **Hypocaloric diet** | ***p*** | **range** |
| --- | --- | --- | --- | --- |
| RBC (x 10^6^/µL) | 5.40 (5.18-5.60) | 5.10 (4.96-5.35) | 0.3 | 3.8-6.68 |
| Hb (g/dl) | 15.80 (15.52-16.00) | 13.60 (12.80-14.50) | 0.013 | 10.4-16.5 |
| MCH (pg) | 29.30 (28.71-30.85) | 19.87 (19.08-21.37) | 0.007 | 18.37-36.98 |
| MCHC (g/dl) | 35.90 (33.76-37.70) | 29.40 (29.00-30.85) | <0.001 | 25.41-80.55 |
| MCV (fl) | 75.20 (72.20-81.40) | 68.50 (67.42-70.75) | 0.017 | 29.41-123.07 |
| PLT (x 10^5^/µL) | 5.24 (5.11-5.40) | 3.97 (3.67-4.50) | 0.003 | 1.7-5.57 |
| PCV (%) | 41.90 (40.30-42.60) | 30.38 (30.00-31.50) | <0.001 | 18-48 |
| WBC (x 10^5^/mm3) | 9.20 (9.10-9.79) | 5.24 (5.07-5.99) | <0.001 | 4.4-14.8 |
| Lymphocytes (%) | 77.90 (72.75-79.06) | 65.80 (62.25-68.35) | 0.001 | 61-86 |
| Neutrophylis (%) | 26.90 (26.20-27.85) | 18.64 (17.90-19.08) | <0.001 | 13-36 |
| Eosinophylis (%) | 2.48 (2.07-2.57) | 1.09 (1.02-1.11) | <0.001 | 0-6 |
| Basophylis (%) | 0.29 (0.28-0.31) | 0.19 (0.18-0.21) | <0.001 | 0-2 |
| Monocytes (%) | 0.19 (0.18-0.20) | 0.15 (0.15-0.17) | 0.019 | 0-1 |
| Glucose (mg/dl) | 148.20 (111.45-161.50) | 80.40 (75.40-90.35) | <0.001 | 62.4-201.8 |
| Total cholesterol (mg/dl) | 92.10 (78.20-97.05) | 44.40 (41.60-49.35) | <0.001 | 14.4-81.7 |
| HDL (mg/dl) | 40.65 (40.32-43.75) | 27.90 (22.63-30.30) | <0.001 | 9.7-42.1 |
| LDL (mg/dl) | 30.42 (29.95-35.07) | 10.22 (10.05-15.76) | <0.001 | 9.66-49.82 |
| TG (mg/dl) | 45.24 (42.77-49.50) | 29.50 (27.90-30.65) | 0.026 | 2.7-47.8 |
| AST (U/L) | 225.60 (217.35-233.35) | 110.30 (107.25-116.90) | <0.001 | 0.2-838.3 |
| ALT (U/L) | 101.20 (98.60-103.80) | 77.90 (75.00-79.55) | 0.002 | 1-223.3 |
| ALP (U/L) | 551.26 (546.28-567.34) | 199.80 (192.65-202.30) | <0.001 | 160.8-838.3 |
| Scr (mg/dl) | 0.75 (0.70-0.81) | 0.25 (0.22-0.31) | 0.002 | 0.2-1.2 |
| BUN (mg/dl) | 38.20 (36.15-40.70) | 19.20 (18.20-19.75) | 0.002 | 17.26-45.12 |
